# Supplementary material for: Iterative improvement in the automatic modular design of robot swarms
Source: PeerJ Comput Sci. 2020 Dec 7;6:e322. doi: 10.7717/peerj-cs.322 (PMC7924708; doi:10.7717/peerj-cs.322)
Supplement: Supplemental Information 3 [file peerj-cs-06-322-s003.zip › argos3/doc/api/standalone/a00466_source.html]

ARGoS: plugins/robots/foot-bot/simulator/footbot\_gripper\_default\_actuator.cpp Source File


- Main Page
- Related Pages
- Namespaces
- Classes
- Files

- File List
- File Members

# plugins/robots/foot-bot/simulator/footbot\_gripper\_default\_actuator.cpp

Go to the documentation of this file.

```
00001 
00007 #include "footbot_gripper_default_actuator.h"
00008 #include <argos3/core/utility/logging/argos_log.h>
00009 #include <argos3/core/simulator/entity/composable_entity.h>
00010 
00011 namespace argos {
00012 
00013    /****************************************/
00014    /****************************************/
00015 
00016    CFootBotGripperDefaultActuator::CFootBotGripperDefaultActuator() :
00017       m_pcGripperEquippedEntity(NULL) {}
00018 
00019    /****************************************/
00020    /****************************************/
00021 
00022    void CFootBotGripperDefaultActuator::SetRobot(CComposableEntity& c_entity) {
00023       m_pcGripperEquippedEntity = &(c_entity.GetComponent<CGripperEquippedEntity>("gripper"));
00024       m_pcGripperEquippedEntity->Enable();
00025    }
00026 
00027    /****************************************/
00028    /****************************************/
00029 
00030    void CFootBotGripperDefaultActuator::Update() {
00031       m_pcGripperEquippedEntity->SetLockState(Abs(m_cAperture) / CRadians::PI_OVER_TWO);
00032    }
00033 
00034    /****************************************/
00035    /****************************************/
00036 
00037    void CFootBotGripperDefaultActuator::Reset() {
00038       Unlock();
00039    }
00040 
00041    /****************************************/
00042    /****************************************/
00043 
00044 }
00045 
00046 REGISTER_ACTUATOR(CFootBotGripperDefaultActuator,
00047                   "footbot_gripper", "default",
00048                   "Carlo Pinciroli [ilpincy@gmail.com]",
00049                   "1.0",
00050                   "The footbot gripper actuator.",
00051                   "This actuator controls the foot-bot gripper. For a complete description of its\n"
00052                   "usage, refer to the ci_footbot_gripper_actuator.h file.\n\n"
00053                   "REQUIRED XML CONFIGURATION\n\n"
00054                   "  <controllers>\n"
00055                   "    ...\n"
00056                   "    <my_controller ...>\n"
00057                   "      ...\n"
00058                   "      <actuators>\n"
00059                   "        ...\n"
00060                   "        <footbot_gripper implementation=\"default\" />\n"
00061                   "        ...\n"
00062                   "      </actuators>\n"
00063                   "      ...\n"
00064                   "    </my_controller>\n"
00065                   "    ...\n"
00066                   "  </controllers>\n\n"
00067                   "OPTIONAL XML CONFIGURATION\n\n"
00068                   "None.\n",
00069                   "Usable"
00070    );
00071
```

---

Generated on 10 Jul 2018 for ARGoS by 
 1.6.1
